# A comparison of Chinese multicenter breast cancer database and SEER database

**DOI:** 10.1038/s41598-022-14573-4

**Published:** 2022-06-21

**Authors:** Yingjie Niu, Fan Zhang, Dong Chen, Guolin Ye, Yong Li, Yong Zha, Wenlin Chen, Dequan Liu, Xiaoming Liao, Qinghua Huang, Wei Tang, Gengxi Cai, Rong Guo, Hongyang Li, Shicong Tang

**Affiliations:** 1 Department of Hepatobiliary and Pancreatic Surgery, The Third Affiliated Hospital of Kunming Medical University, Yunnan Cancer Hospital, Kunming, Yunnan People’s Republic of China; 2 Department of Breast Surgery, The Third Affiliated Hospital of Kunming Medical University, Yunnan Cancer Hospital, Kunming, Yunnan People’s Republic of China; 3 Department of Breast Surgery, Affiliated Tumor Hospital of Guangxi Medical University, Nanning, Guangxi People’s Republic of China; 4 Department of Ultrasound, Caner Hospital of Yunnan Province, The Third Affiliated Hospital of Kunming Medical University, Kunming, 650118 Yunnan People’s Republic of China; 5 Department of Breast Surgery, The First People’s Hospital of Foshan, #81, North Lingnan Avenue, Chancheng, Foshan, Guangdong People’s Republic of China

**Keywords:** Cancer, Cancer

## Abstract

There are different characteristics of BC in developing countries and developed countries. We intended to study the factors which influence the survival and prognosis of BC between southern China and the United States. (**a**) To study the two groups BC patients in southern China from 2001 to 2016 and SEER database from 1975 to 2016. (b) To register, collect and analyze the clinicopathological features and treatment information. Our study found that there are significant differences in tumor size, positive lymph node status and KI-67 between southern China and SEER cohort (*P* < 0.000). The positive lymph node status may be one of the causes of difference of morbidity and mortality of BC patients in China. Furthermore, the differences in treatment methods may also account for the differences between China and seer databases.

## Introduction

Breast cancer (BC) is one of the most common cancers of women in the world, which is also the second main cause of cancer death in women. There are about 1.5 million women (25% of all cancer women) are diagnosed with BC every year all around the word, and it is estimated to increase to 2.2 million per year by 2025^1–3^. However, there are literatures showed that the mortality of BC in high-income countries is declining, while it is increasing in low and middle income countries^4^.

It is reported that, the incidence of BC in China is expanding at twice the rate of the world, which has risen from 23.37/10 million in 2007 to 28.42/10 million in 2013^5,6^. In addition, there are researches showed that the incidence rate of BC is increasing in China, and it may reach 2 million 500 thousand cases by 2021, however, its survival rate is low, the 5-year survival rate ranged from 40 to 60%^7,8^. Then, a lot of research suggests that there is a large difference of BC between China and other regions, which may due to the difference of clinicopathological features, the details as follows^9–11^. Jarzab et al. considered that the prognosis of lumen type G1 tumor is good, while G3 tumor is poor in BC of China^12^. Meanwhile, Zhang et al. reported that, lymph node metastasis seriously affects the prognosis of BC patients. In addition, the expression of KI-67 and prognosis were closely related to pTNM stage and PR expression^13^. It is indicated that Ki-67 positive can lead to higher histological grade of BC^14^. However, HER2 overexpression can lead to invasive breast cancer, which overexpression was negatively correlated with the expression of PR and ER^15^.

In contrast, there are about 12% of women in the United States are diagnosed with BC in their lifetime, nevertheless, it is estimated that there are about 3.1 million BC survivors each year^16,17^. With the development of treatment strategies, the mortality of BC has been decreased in the United States, which the 5-year survival rate was about 90% after treatment^18^. A large number of studies have shown that, BC also has its own characteristics in the United States. DeSantis CE et al. reported that BC patients in the United States from 2004 to 2014, young women have higher invasive and specific genomic characteristics, meanwhile, the incidence of HR positive (ER positive or PR positive) BC increased, while the morbidity of HR negative tumors decreased^19–21^.

There are different researchers in the world comparing BC in China and other regions, and finding that there exist some differences between them. However, the comparison of BC patients between southern China and the United States has not been reported. This study aims to investigate the differences of BC patients between China and the population-based Surveillance, Epidemiology, and End Results (SEER) cohort. In addition, our study intends to examine the age, stage and grade of tumor, ER, PR, HER2, KI-67 and treatment methods, in order to analyze the age distribution, clinical characteristics, treatment and prognosis of BC patients in Chinese multicenter breast cancer database and SEER database, so that we could compare the two groups.

## Methods

### Patients and ethics

**Figure 1:**
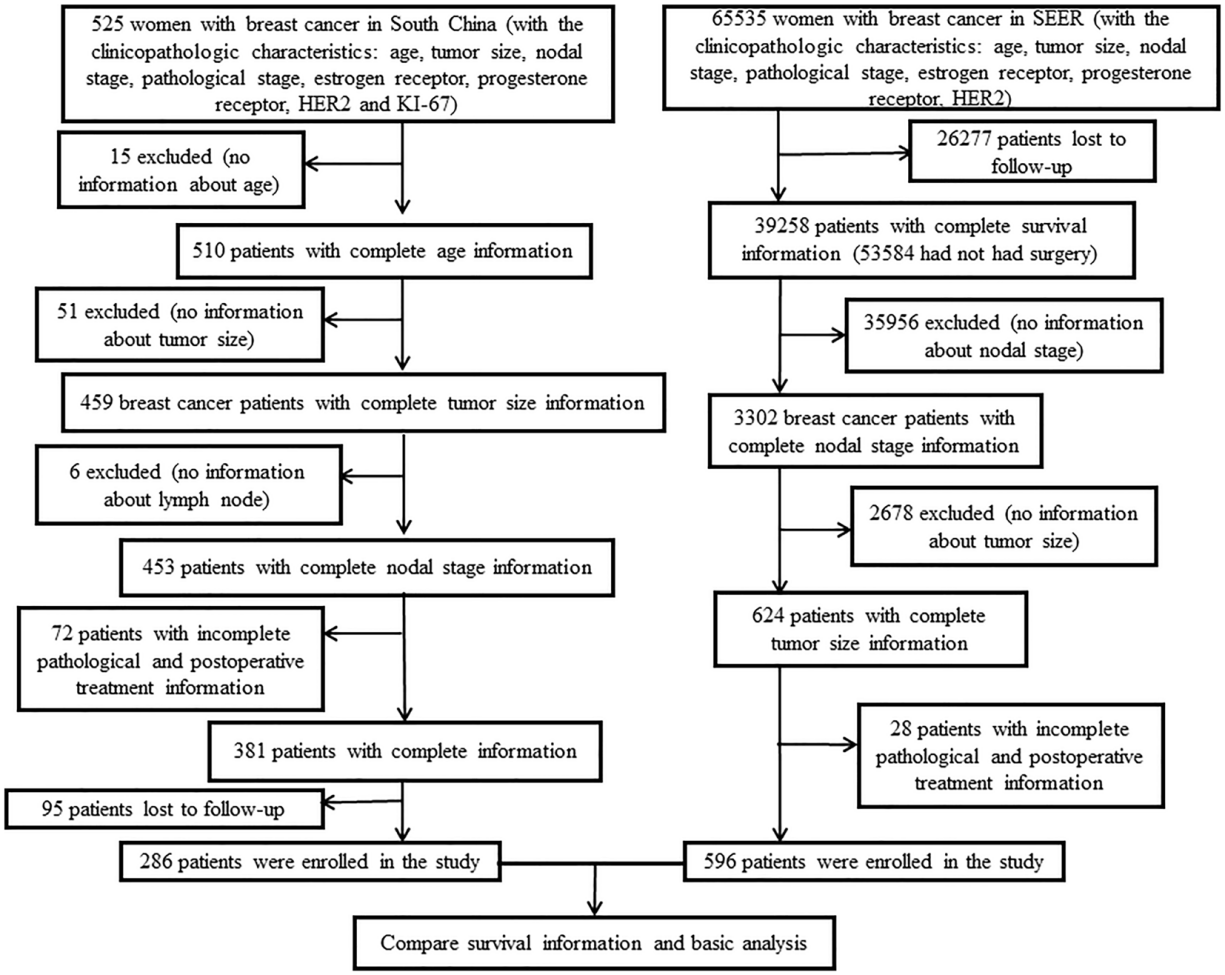
Flow chart for breast cancer patients in both Southern China and SEER database.

We conducted a retrospective analysis and comparison of the patients who have been diagnosed with primary breast cancer in southern China (2001–2016) and SEER database (1975–2016). Overall, there was a total of 525 breast cancer patients were diagnosed in southern China, among them, 15 patients were excluded from this study due to lack of age information. In addition, 129 patients were removed, which without tumor stage, ER, PR, HER2, KI-67 and treatment information. Additionally, there are about 95 patients were lost. Finally, a total of 286 patients were included in the study (Fig. 1). The study was approved by Institutional Review Board of Yunnan Cancer Hospital, Cancer Hospital Affiliated to Guangxi Medical University and Foshan first people's Hospital. Informed consent was obtained from all individual participants included in the study. All procedures implemented in studies involving human participants were in accordance with the ethical standards of the institutional and/or national research committee, and with the 1964 Helsinki Declaration and its later amendments or comparable ethical standards. The SEER cohort was derived from the SEER database (November 2018 submission) by using SEER*Stat software provided by the National Cancer Institute (NCI). There were 65,535 breast cancer patients, among them, 26,277 cases were lost. Except for without complete information (age, stage and grade of tumor, ER, PR, HER2, KI-67 and treatment) patients, which was about 38,662 cases, there were 596 patients included in this study (Fig. 1).

### Clinical data collection

A retrospective review of medical records and pathology reports was conducted. Staging was performed according to the American Joint Committee on Cancer (AJCC) guidelines^22^. The age of the patients was classified to young adult group (< 40), middle aged group (40–70) and aged + group (> 70), and then, calculated the median age of each age group (35, 48, 75), finally, statistical analysis was carried out respectively. *A* cutoff of 14% for KI-67 was used, which was recommended by 2011 St Gallen consensus panel^23^, and then*,* we divided the group of KI-67 ≥ 14% into two subgroups, according to the median (51.7% in southern China)*.* Patients in southern China were told to have an examination and treatment according to the guidelines of the breast cancer center and were followed up by telephone, and collect the information about survival and treatment, including date of progression metastasis, date of relapse and date cause of death.

### Statistical methods

The IBM SPSS Statistics (Version 21.0; IBM Corp., New York, USA) and GraphPad Prism (Version 6.0; GraphPad software, Inc., LaJolla, CA, USA) were used for statistical analysis. Disease-free survival (DFS) was measured from the beginning of the operation to the first recurrence/metastasis of the tumor or the death of the subject for any reason (the last follow-up time was the patients who lost the follow-up; the patients who were still alive at the end of the study were the end of the follow-up). Overall Survival (OS) was measured from the beginning of operation to death due to any reason. Univariate analysis and multivariate analysis was performed by Cox regression analysis, according to comparing the age (< 40 and ≥ 40), tumor size (≤ 2 cm and > 2 cm), node status, ER, PR, HER2, KI-67, surgery and radiation. Kaplan–Meier method was used to estimate DFS and OS, log-rank test was used to compare the patients with different clinicopathologic characteristics. The count data were tested by χ^2^ test, fisher exact probability method was used when the cases was less than 6. Statistical significance was set at a *P* < 0.05, *P* < 0.01 had significant difference.

### Ethics approval and consent to participate

All procedures implemented in studies involving human participants were in accordance with the ethical standards of the institutional and/or national research committee, and with the 1964 Helsinki Declaration and its later amendments or comparable ethical standards. Ethics/Institutional Review Board approval of research—Yunnan Cancer Hospital, Cancer Hospital Affiliated to Guangxi Medical University and Foshan first people's Hospital. Informed consent was obtained from all individual participants included in the study.

### Consent for publication

All patients enrolled in the study signed the consent for publication.

## Results

### Patients

Between 2001 and 2016, a total of 525 females received surgical treatment in the southern China. Among them, 15 patients without the age information, so they were excluded from this study. There were 51 patients without the information of tumor size, 6 patients without node status, and in the left 453 cases, 75 patients without pathological stage and treatment information and 95 patients were lost, all in all, there were 286 patients included in the southern China cohort. Between 1975 and 2016, a total of 65,535 patients were included in SEER database, among them, 26,277 patients were lost to follow-up, 35,956 patients without the information of lymph node stage, 2678 cases had no tumor size information, 28 patients had no complete information of tumor staging and treatment, so there were about 596 patients included from the SEER cohort (Fig. 1).

### Comparison of clinicopathological features between southern China and SEER cohort

All patients were divided into three subgroups: young adult group (< 40), middle aged group (40–70) and aged + group (> 70). Among 510 breast cancer patients in southern China, 101 (19.8%) patients were under 40, what’s more, middle aged patients account for the most, about 396 (77.65%) patients. Between 1975 and 2016, there were 65,535 BC patients were included SEER database, the proportion of young patients was slightly less than that in southern China, was 4024 (6.14%), but there was no statistical significance between the two (*P* = 0.923). However, the middle aged patients account for 41,256 (62.95%) in SEER cohort (*P* = 0.000). There was a difference between southern China and SEER cohort, the proportion of aged + group was higher in SEER cohort, which was about 13,104 (20.00%), it was significantly higher than 5 patients (0.98%) in southern China, which was statistically significant (*P* = 0.048) (Tab. 1). BC patients in southern China and SEER cohort were compared, the age of newly diagnosed cases in the two groups was 40–70 years old. While, there was a different between the two groups: BC patients in the subgroup of 40–48 years old (48 years old was the median age of 40–70 years old) were roughly similar to the subgroup of 48–70 years old in southern China, there were 194 (38.04%) patients and 202 (39.61%) patients, respectively. However, BC patients in SEER cohort were slightly older, it was 9105 (13.89%) patients in the subgroup of 40–48 years old, while there were 32,151 (49.06%) patients in the subgroup of 48–70 years old (Table 1).

**Table 1:** Clinicopathologic characteristics of breast cancer patients in Southern China and SEER cohorts.

Patient characteristics	Southern China	SEER	χ^2^-value	P value
	N (%)	N (%)		
**Age at diagnosis (year)**			0.009	0.923
Young adult group (< 40)				
< 35	38 (7.45)	1495 (2.28)		
≥ 35	63 (12.35)	2529 (3.86)		
Middle aged group (40–70)			163.919	0.000
≤ 48	194 (38.04)	9105 (13.89)		
> 48	202 (39.61)	32,151 (49.06)		
Aged + group(> 70)			0.077	0.048
≤ 75	8 (1.57)	7151 (10.91)		
> 75	5 (0.98)	13,104 (20.00)		
Tumor size(cm)			29.923	0.000
Tx	10 (2.01)	51 (0.08)		
Tis	1 (0.20)	1 (0)		
Invasive group				
≤ 2	79 (15.86)	934 (1.43)		
> 2 ≤ 5	323 (64.86)	367 (0.56)		
> 5	46 (9.24)	63 (0.1)		
Missing	39 (7.83)	64,071 (97.8)		
Nodal stage			20,387.140	0.000
pN0, no nodal metastasis	206 (40.39)	1145 (1.75)		
pN1, 1–3 nodal metastasis	132 (25.88)	222 (0.34)		
pN2, 4–9 nodal metastasis	76 (14.90)	34 (0.05)		
pN3, ≥ 10 nodal metastasis	49 (9.61)	40 (0.06)		
Unknown	47 (9.22)	64,094 (97.80)		
Pathological stage			25.662	0.000
0–1	97 (19.02)	7082 (10.82)		
2	262 (51.37)	14,936 (22.81)		
3	133 (26.08)	12,993 (19.75)		
4	18 (3.53)	1255 (1.92)		
Unknown	0 (0)	29,269 (44.7)		
Estrogen receptor			106.162	0.000
Negative	178 (34.9)	5563 (17.96)		
Positive	290 (56.86)	20,233 (65.34)		
Missing	42 (8.24)	5172 (16.70)		
Progesterone receptor			30.794	0.000
Negative	182 (35.69)	8110 (26.2)		
Positive	270 (52.94)	179,194 (55.55)		
Missing	58 (11.37)	5646 (18.24)		
HER2-positive			611.595	0.000
Negative	165 (32.35)	1530 (85.62)		
Positive	283 (55.49)	169 (9.46)		
Missing	62 (12.16)	88 (4.92)		
Ki-67(%)			NA	NA
< 14	100 (19.61)	NA (NA)		
< 51.7 median	170 (33.33)	NA (NA)		
≥ 51.7 median	95 (18.63)	NA (NA)		
Missing	145(28.43)	NA (NA)		

According to TNM stage, BC patients were divided into Tx, Tis and invasive group (including ≤ 2 cm, > 2 ≤ 5 cm, > 5 cm), among them, invasive group account for the most, there were 448 (89.96%) patients in southern China and 1364 (2.99%) patients in SEER cohort, *P* = 0.000. In southern China, the tumor size was 2–5 cm accounted for the most, were 323 (64.86%) patients. However, there were too many data were missing, were 64,071 (97.8%) cases, Tis subgroup in the two was both 1 case (Table 1). There were 257 (50.39%) patients with node metastasis in southern China and 296 (0.45%) cases in SEER cohort. Among the two groups, no lymph node metastasis accounted for the most, were about 206 (40.39%) patients and 1145 (1.75%) patients. While, there were too many data of lymph node status missing, were 29,269 (44.7%) patients (Table 1). Comparing southern China and SEER database, there was statistical significance in tumor stage (*P* = 0.000). Among them, the proportion of stage 2 was the highest, were 262 (51.37%) cases and 14,936 (22.81%) cases respectively. Next, it was stage 3, were 133 (26.08%) cases and 12,993 (19.75%) cases. However, there were many missing data about tumor stage in SEER database, were about 29,269 (44.7%) cases (Table 1). The expression of ER was counted in both southern China and SEER cohort (*P* = 0.000). Among them, ER (+) accounted for a higher proportion, were 290 (56.86%) cases and 20,233 (65.34%) cases respectively, and ER (−) were 178 (34.9%) cases and 5563 (17.96%) cases, respectively (Table 1). Similarly, the differences in expression of PR in southern China and SEER cohort was also statistically significant (*P* = 0.000). Among them, PR (+) was higher in both southern China and SEER cohort, were 270 (52.94%) cases and 179,194 (55.55%) cases, PR (−) were 182 (35.69%) cases and 8110 (26.2%) cases (Table 1). While, the expression of HER2 was different between the two groups (*P* = 0.000). The expression of HER2 (+) accounted for the most in southern China, was 283 (55.49%) cases, but there was only 169 (9.46%) cases in SEER cohort, with the proportion of PR (−) was high, was 1530 (85.62%) cases (Table 1). Additionally, the expression of KI-67 was different in the two groups, there were 365 (71.57%) cases of KI-67 (+), among them, it was the most between 14 and 51.7%, was 170 (16.58%) cases. However, there was no data about the expression of KI-67 in SEER cohort (Table 1).

### Survival and prognosis analysis

**Figure 2:**
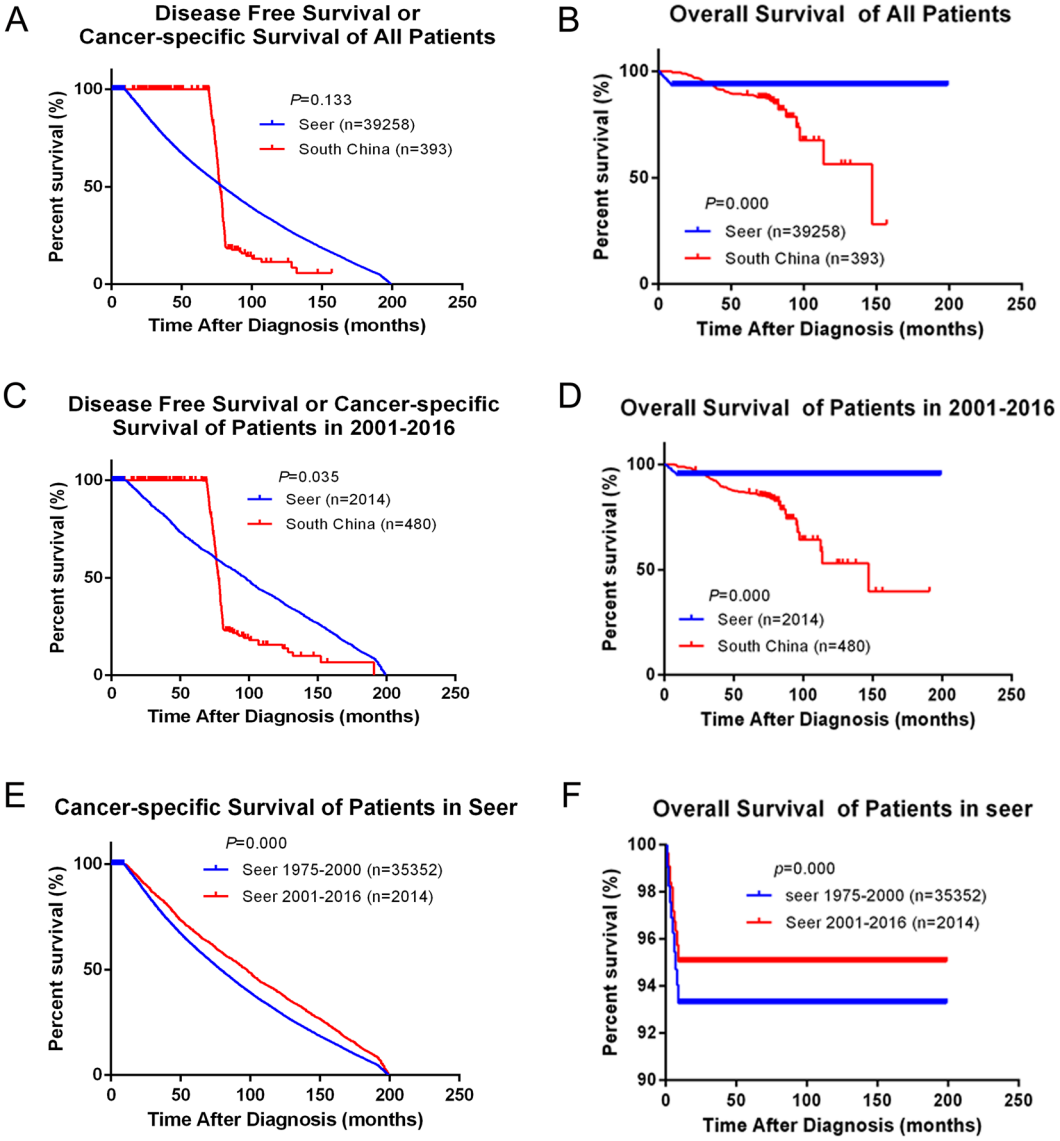
Comparison of all patients’ DFS, CSS and OS in Southern China and SEER database. (**A**,**B**) Kaplan–Meier estimates disease-free survival (DFS), cancer-specific survival (CSS) and overall survival (OS) of all patients in both Southern China and SEER database, (**C**,**D**) Kaplan–Meier estimates DFS or CSS and OS of patients in 2001–2016 in both Southern China and SEER database, (**E**,**F**) Kaplan–Meier estimates DFS or CSS and OS in both 1975–2000 and 2001–2016 in SEER database.

Between 2001 and 2016, there were 393 BC patients who were followed up in southern China. Meanwhile, between 1975 and 2016, 39,258 BC patients with complete follow-up information were analyzed. During the follow-up period, there were 59 patients died, 219 patients were alive and 115 patients relapsed in southern China. While in SEER cohort, there were 3613 patients died and 35,645 patients were alive. DFS, cancer-specific survival (CSS) and OS of all included breast cancer patients were compared: in comparing of DFS or CSS, there was no significant different between southern China and SEER cohort (*P* = 0.133), but there was significant difference in OS (*P* = 0.000), and the OS in SEER cohort. It was significantly higher than southern China (Fig. 2A,B). Secondly, since the data of southern China was only included from 2001 to 2016, a layered statistic was used to count the DFS or CSS and OS in both southern China and SEER database from 2001 to 2016. Among them, in the first 70 months of follow-up, DFS in southern China was higher than CSS in SEER cohort, and then, CSS in SEER cohort was significantly higher than that in southern China (*P* = 0.035), and OS in this period also has significant statistical different (*P* = 0.000), SEER cohort was significantly higher than that in southern China (Fig. 2C,D). Finally, SEER cohort was analyzed in stages, dividing into 1975–2000 and 2001–2016 two subgroups, furthermore, CSS and OS of each subgroup were counted respectively. The results showed that, in SEER cohort, the DFS or CSS and OS of 2001–2016 were significantly higher than 1975–2000 (*P* = 0.000) (Fig. 2E,F).

**Figure 3:**
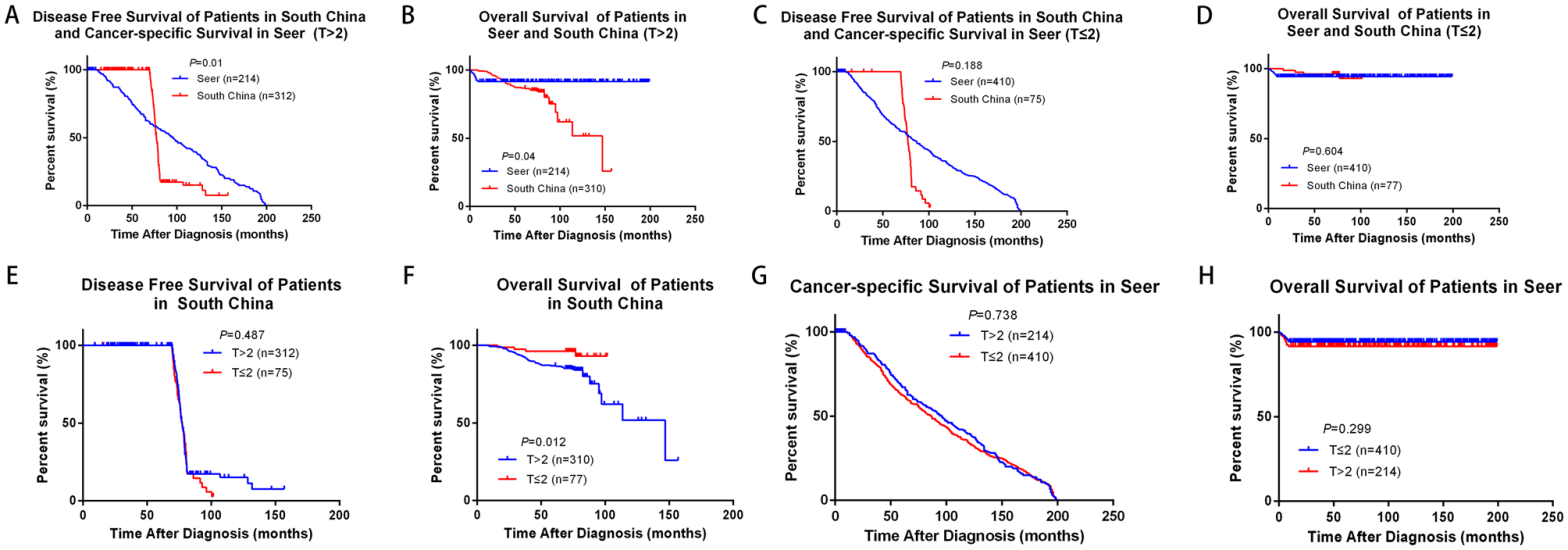
Comparison of different tumor size in Southern China and SEER database. (**A**,**B**) Kaplan–Meier estimates DFS or CSS and OS of patients whose T > 2 cm in both Southern China and SEER database, (**C**,**D**) Kaplan–Meier estimates DFS or CSS and OS of patients whose T ≤ 2 cm in both Southern China and SEER database, (**E**,**F**) Kaplan–Meier estimates DFS and OS of different tumor size in Southern China, (**G**,**H**) Kaplan–Meier estimates CSS and OS of different tumor size in SEER database.

**Figure 4:**
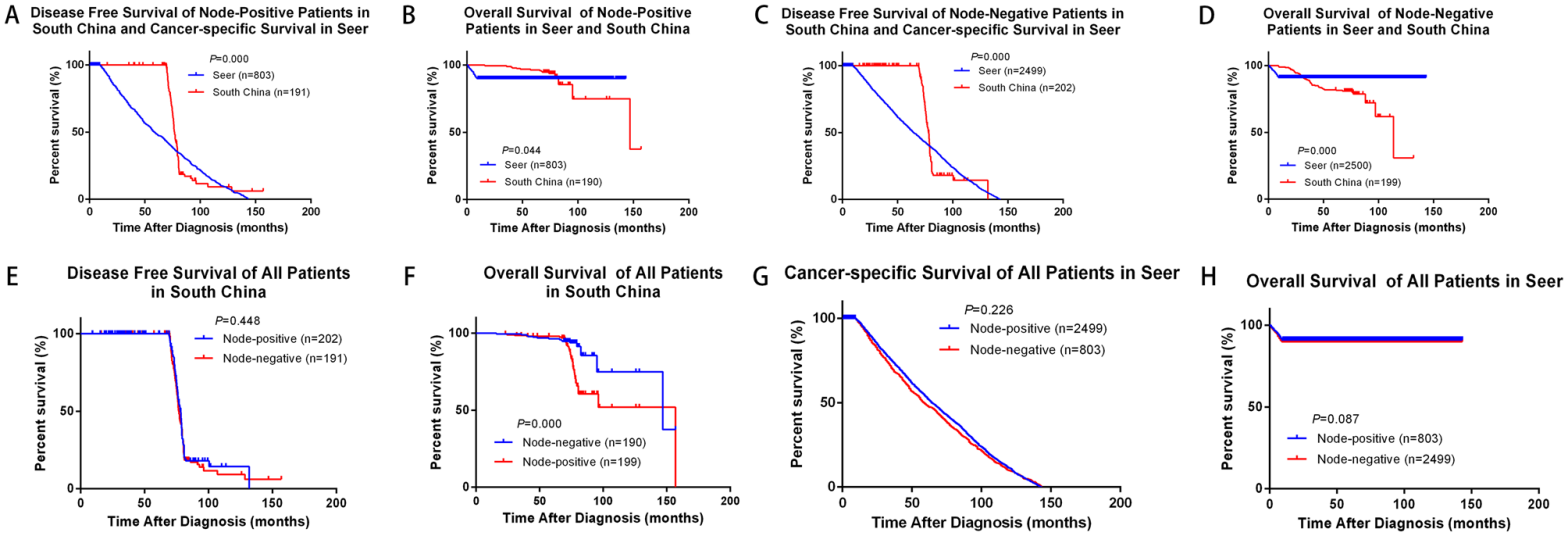
Comparison of different Nodal stage in Southern China and SEER database. (**A**,**B**) Kaplan–Meier estimates DFS or CSS and OS of Node-positive patients in both Southern China and SEER database, (**C**,**D**) Kaplan–Meier estimates DFS or CSS and OS of Node-negative patients in both Southern China and SEER database, (**E**,**F**) Kaplan–Meier estimates DFS and OS of different nodal stage in Southern China, (**G**,**H**) Kaplan–Meier estimates CSS and OS of different nodal stage in SEER database.

**Figure 5:**
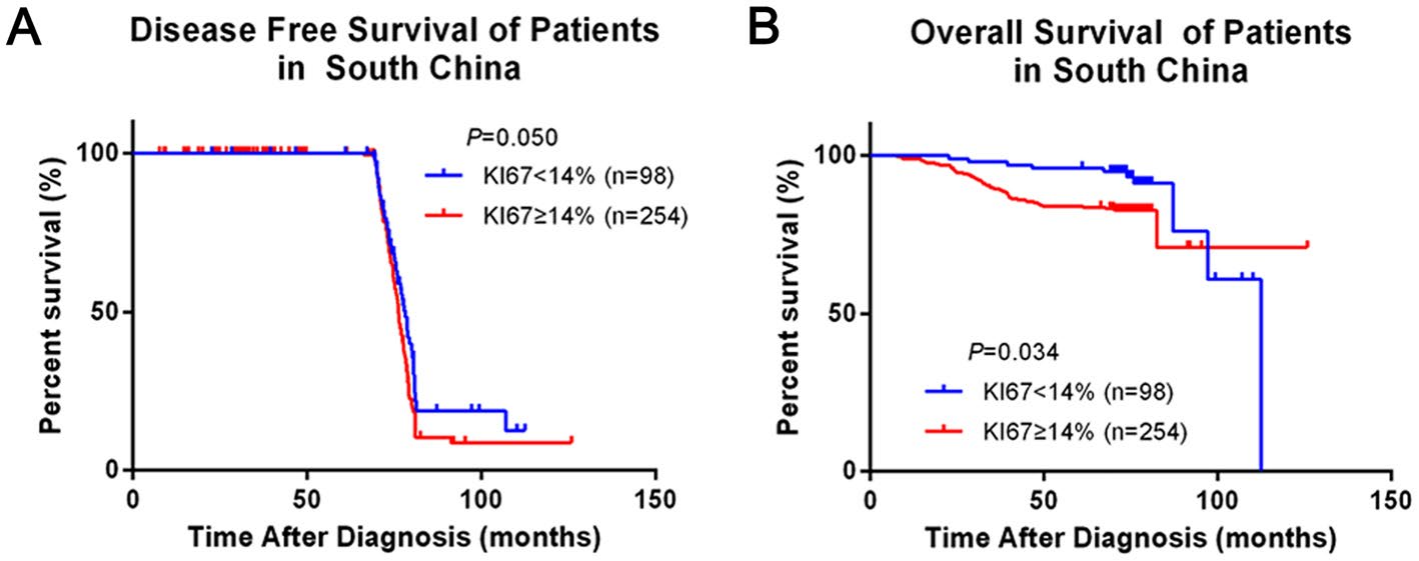
Comparison of different KI-67 expression in Southern China. (**A**) Kaplan–Meier estimates DFS of different KI-67 expression in Southern China, (**B**) Kaplan–Meier estimates OS of different KI-67 expression in Southern China.

We analyzed the influence of different clinicopathological features on survival and prognosis of BC patients in southern China. By analyzing the effects of age, tumor size, lymph node status, ER, PR, HER2, KI-67, surgery and radiotherapy on the prognosis of breast cancer, we found that tumor size, positive lymph node status and KI-67 expression affected OS of BC patients in southern China, which showed significant statistical difference (*P* = 0.018, *P* = 0.000, *P* = 0.034 respectively) (Supplementary Fig. 1). We further analyzed and compared the effect of different tumor size on survival of different BC cohorts. There were statistical differences of DFS or CSS and OS in SEER cohort and southern China when the tumor size (T) > 2 cm (*P* = 0.01 and *P* = 0.04), however, DFS or CSS and OS were not statistically different in the two groups when T ≤ 2 cm (*P* = 0.188 and *P* = 0.604) (Fig. 3A,D). Secondly, the effects of different tumor sizes on the survival of BC patients in each cohort were analyzed separately. Among them, tumor size had little effect on DFS in southern China (*P* = 0.487), but for OS, there was significant statistical difference, OS in T > 2 cm group was significantly lower than T ≤ 2 cm (*P* = 0.012) (Fig. 3E,F). However, for SEER cohort, CSS and OS of T > 2 cm group were slightly lower than that of T ≤ 2 cm group, but there was no statistical difference (*P* = 0.738 and *P* = 0.299) (Fig. 3G,H). We analyze and compared the effect of different node stage on survival of different BC cohorts. Positive-node affected DFS or CSS and OS in both southern China and SEER cohort (*P* = 0.000 and *P* = 0.044). Meanwhile, negative-node also affected DFS or CSS and OS in the two groups (*P* = 0.000 and *P* = 0.000). OS of SEER cohort with different lymph node status was higher than that of southern China (Fig. 4A–D). Analyzing southern China and SEER cohort separately, DFS or CSS and OS of positive-node were lower than negative-node, among them, OS of lymph node status has significant statistical difference (*P* = 0.000), but DFS or CSS of lymph node status has no statistical difference (*P* = 0.448) (Fig. 4E,F). But fcer SEER cohort, CSS and OS of positive-node was slightly higher than negative-node, while there was no statistical difference (*P* = 0.226 and *P* = 0.087) (Fig. 4G,H). We analyzed and compared the effect of expression of KI-67 on survival of southern China. Among the subjects included in this study, DFS and OS of KI-67 < 14% both higher than ≥ 14%, there were significant statistical difference (*P* = 0.05 and *P* = 0.034) (Fig. 5A,B). Multivariate analysis and univariate analysis of southern China and SEER cohort was performed by Cox regression analysis. In univariate analysis of DFS, T > 2 cm, positive-node, ER (+), PR (+), HER2 (+), surgery and radiation all had no significant influence on the increased risk of death. Among them, the hazard ratio (HR) of KI-67 high expression group was 1.376, 95% CI 1.000–1.894, *P* = 0.050. However, in multivariate analysis of DFS, all the clinicopathological features of the included studies were statistically significant (Table 2).

**Table 2:** Cox regression analysis for disease-free survival in southern China.

Variables	Univariate analysis			Multivariate analysis		
	Hazard ratio	95% confidence interval	P value	Hazard ratio	95% confidence interval	P value
Age	0.874	0.644–1.186	0.388	0.977	0.600–1.591	0.926
Tumour stage > 2 cm	0.894	0.652–1.227	0.488	1.257	0.793–1.990	0.330
Node-positive	1.108	0.850–1.446	0.448	1.116	0.754–1.653	0.584
Estrogen receptor-positive	1.109	0.846–1.454	0.453	1.154	0.660–2.017	0.615
Progesterone receptor-positive	1.116	0.852–1.463	0.426	1.122	0.662–1.904	0.669
HER2-positive	0.885	0.675–1.162	0.380	1.091	0.740–1.608	0.662
Ki-67 high expression	1.376	1.000–1.894	0.050	0.960	0.385–2.392	0.930
Mastectomy	0.934	0.521–1.673	0.818	0.985	0.650–1.492	0.943
Radiation therapy	1.016	0.770–1.342	0.910	0.889	0.596–1.326	0.564

Multivariate analysis and univariate analysis of southern China was performed by Cox regression analysis. In the univariate analysis of OS, it was significantly associated with increased risk of death and T > 2 cm (HR 3.406, 95% CI 1.232–9.417, *P* = 0.018), positive-node status (HR 0.308, 95% CI 0.169–0.564, *P* = 0.000) and KI-67 high expression (HR 2.128, 95% CI 1.057–4.285, *P* = 0.034). In the multivariate analysis of OS, positive-node status (HR 0.226, 95% CI 0.098–0.519, *P* = 0.000) was significantly associated with increased risk of disease survival (Table 3).

**Table 3:** Cox regression analysis for overall survival in southern China.

Variables	Univariate analysis			Multivariate analysis		
	Hazard ratio	95% confidence interval	P value	Hazard ratio	95% confidence interval	P value
Age	0.771	0.462–1.288	0.321	0.836	0.386–1.814	0.651
Tumour stage > 2 cm	3.406	1.232–9.417	0.018	0.710	0.246–2.049	0.526
Node-positive	0.308	0.169–0.564	0.000	0.226	0.098–0.519	0.000
Estrogen receptor-positive	0.716	0.454–1.127	0.149	1.888	0.702–5.079	0.208
Progesterone receptor-positive	0.647	0.404–1.035	0.069	0.561	0.207–1.518	0.255
HER2-positive	0.945	0.599–1.490	0.808	1.576	0.825–3.009	0.168
Ki-67 high expression	2.128	1.057–4.285	0.034	1.013	0.115–8.902	0.990
Mastectomy	3.351	0.463–24.256	0.231	1.020	0.501–2.077	0.957
Radiation therapy	1.088	0.677–1.748	0.727	1.130	0.584–2.188	0.717

### Comparison of treatment

There were differences of treatments between southern China and SEER cohort (Table 4). We analysed both of the two databases from 2001 to 2016, and there were 389 (97.01%) patients received chemotherapy in southern China, but there were 1574 (26.64%) patients in SEER cohort have received chemotherapy (P = 0.000). However, the treatment of surgery was similar, there were 387 (95.09%) patients performed mastectomy in southern China cohort (including simple resection and modified radical operation), and for SEER cohort, there were 5524 (93.50%) patients had breast surgery (the specific operation method is not clear). However, it was the same of the two about whether to receive radiotherapy or not, and there was a significant statistical different (*P* < 0.001). Among them, there were 351 (72.82%) patients had not performed radiotherapy in southern China, with 2223 (37.63%) patients in SEER cohort.

**Table 4:** Treatment of breast cancer patients in Southern China and SEER cohorts.

Treatment	Southern China	SEER	χ^2^-value	P value
	N (%)	N (%)		
**Chemotherapy**			867.474	< 0.001
Yes	389 (97.01)	1574 (26.64)		
No	12 (2.99)	4334 (73.36)		
**Breast surgery**			NA	NA
Breast-conserving surgery	20 (4.91)	5524 (93.50)		
Mastectomy	387 (95.09)			
No breast surgery	0 (0)	384 (6.50)		
**Radiation therapy**			229.469	< 0.001
Yes	131 (27.18)	3685 (62.37)		
No	351 (72.82)	2223 (37.63)		

### Changes in morbidity with years

**Figure 6:**
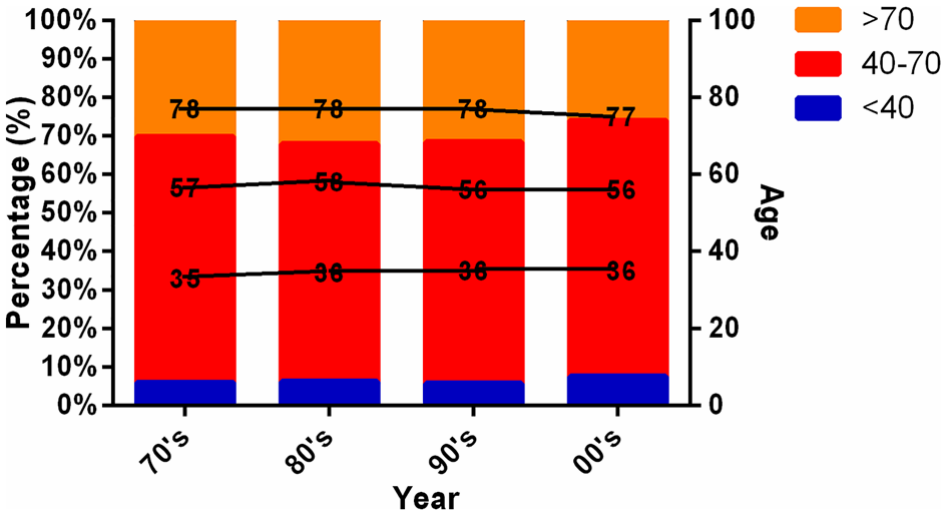
Comparison of different years all patients’ age in SEER database.

We further analyzed and compared the age distribution of BC patients in different years in SEER cohort. Among them, except for 90’s, the proportion of BC patients in young adult group (< 40 years old) showed an increasing trend with age. They were respectively: 70’s: 6%, 80’s: 6.31%, 90’s: 5.76%, 00’s: 7.62%, and the median age was 35 year, 36 year, 36 year and 36 year. In all age groups, the incidence of middle-aged group (40–70 years old) was the highest, followed by 63.89%, 61.74%, 62.67% and 66.42%, and the median age was 57 year, 56 year, 56 year and 56 year. In addition, we found that the morbidity of aged + group (> 70) showed a decreasing trend year by year, respectively was 30.11%, 31.95%, 31.57% and 25.96%, and the median age was orderly 78 year, 78 year, 78 year and 77 year (Fig. 6).

## Discussion

Our research is a very important one, which is the first to analyze and compare the related factors of survival and prognosis of BC patients in southern China and SEER cohort. We analyzed the multiple factors: including age, tumor stage and grade, ER, PR, HER2, KI-67, surgery and radiotherapy, which may influence the survival and prognosis of BC. It is a multi-regional and big data clinical study.

In this study, by comparing and analyzing the age of both southern China and SEER cohort, we found that in southern China from 2001 to 2016, there was about 19.8% BC patients were under 40 year, which was the same as the results of Wang, who had reported that the incidence of young BC patients in China is about 21.97%^24^. However, the morbidity of young BC patients in China is significantly higher than that in western countries (about 4–6%)^25–28^, which was similar to the incidence of our study: it was 6.14% of SEER cohort from 1975 to 2016. All of these suggested that the incidence of BC in China is younger than that in western countries, which indicated that age may be a factor affecting the survival and prognosis of BC patients in southern China and the United States.

To further study DFS and OS, we focused on T stage, positive lymph node status, ER, PR, HER2, KI-67 expression of BC patients, and thought that T stage, positive lymph node status and KI-67 expression all could be regarded as factors, which affected the survival and prognosis of BC patients. Other scholars had also studied tumor stage, and found that there were about 60–70% of BC patients were diagnosed with stage 1, which was higher than Asian countries, but there were only about 10% women were stage 4^29^. This research was similar to our results, our finding showed that most BC patients in southern China from 2001 to 2016 could be diagnosed at early time, among them, there were about 64.86% of patients with T2 BC, 40.39% of patients with N0, 51.37% of patients with stage 2. However, the early diagnosis rate of BC in China is far lower than that in the United States. It showed that in SEER cohort, there were about 65.96% of patients with T1, 79.46% of patients with N0 and 41.18% of patients with stage 2. Cox regression analysis showed that T stage and positive lymph node status were important factors affecting OS of BC. Therefore, we further to prove that stages and grades of tumor had a significant impact on the survival and prognosis of BC. Meanwhile, China should to further strengthen the early diagnosis and treatment of BC, so as to improve the prognosis of BC patients in China.

We further explored the effects of ER, PR, HER2, KI-67 expression on survival and prognosis of BC. The proportion of ER (+) BC patients was similar in both southern China and SEER cohort. It was 56.86% in southern China and 65.34% in SEER cohort, which was slightly lower than that had been reported (about 70%)^30^. It may be related to excessive data deletion in SEER cohort, which was about 16.7% of ER data were missing in this study. Other studies have showed that the most important factors affecting the prognosis of BC were tumor grade and ER status^31^. However, in our study, ER was not an indicator of survival and prognosis of BC. Additionally, there were different treatment methods of BC according to the different status of hormone receptor (HR). Endocrine therapy could be used for ER or PR positive patients, but the effect of chemotherapy was not as good as these of negative patients, and the different treatment methods could significantly affect the prognosis of BC. PR was also an important factor affecting the prognosis of BC. In our study, there was 52.94% of PR positive in southern China, and 55.55% of that in SEER cohort, there was significant statistical difference between the two groups (*P* = 0.000). Similarly, the results of Cox regression analysis showed that PR was also not an indicator of survival and prognosis of BC, which may had a relationship between a large data missing. In addition, Ding et al. found that BC with HER2 and KI-67 overexpression had higher lymph node metastasis rate and higher AJCC tumor stage^32,33^, which was similar to our results. In this study, HER2 positive were 55.49% and 9.46% in southern China and SEER cohort respectively (*P* = 0.000), which was consistent with the literature showing that BC cells from young patients are more likely to show HER2 positive expression^24^. KI-67 positivity was 70.01% in southern China, a high expression is an important factor affecting OS in BC patients. However, DFS was detected by χ^2^ test when KI-67 was regarded as an independent factor, *P* = 0.05, but we believe that this was mainly due to the small sample size, the trend in the conclusion was still valid, as the sample size continues to increase, the value of *P* may gradually decrease. In summary, our results showed that positive-node status was an important factor affecting the prognosis of BC, which also reflected that BC patients in southern China and the United States have different biological behaviors and pathogenesis.

Additionally, the treatment methods of BC were also important factors affecting its prognosis. At present, the main treatments of BC were surgery, radiotherapy, chemotherapy, targeted therapy and hormone therapy^34,35^. Among them, surgery can significantly reduce the mortality rate, which is the most critical step in the treatment of breast cancer, there are five common surgical methods: breast conserving surgery (BCS), simple mastectomy (SM), modified radical mastectomy (MRM), radical mastectomy (RM) and extensive radical mastectomy (ERM)^36^. Among them, Bartelink et al. reported that BCS has the equivalence with mastectomy^37^. However, the comparison of treatment methods between southern China and the United States has not yet reported, we had studied this for the first time. In this study, BC patients enrolled in the study in southern China all underwent surgery, and the treatment including BCS and mastectomy (including SM and MRM). Among them, there were about 95.09% of BC patients performed mastectomy and 4.91% of that performed BCS, and the implementation rate of BCS was significantly lower than that of developed countries, which was similar to the results of Gupta A: the highest implementation rate of BCS in China is only 8.6%^38^. However, there were only 18.24% of BC patients performed mastectomy in SEER cohort, 81.76% of that had not. While, there was no significant effects on DFS and OS for whether performing surgery, which suggesting that surgery had little effect on the survival and prognosis of BC. But for SEER cohort had not include the details of surgical procedures, so its’ effects on survival and prognosis of BC were not studied and analyzed, which may be a potential factor. In recent years, a number of large randomized trials have shown that radiotherapy could significantly reduce the local recurrence of BC, so as to improve the breast preservation rate and obtain good survival rate^38^. However, in this study, the radiotherapy rate of BC patients in southern China and SEER cohort were both not high, were 27.18% and 34.22% respectively, and there were significant statistical different between the two (*P* = 0.001). Nevertheless, the results of Cox regression analysis showed that radiotherapy had no significant effect on both DFS and OS, which may be related to a low radiotherapy rate, but it should be further studied and analyzed. Besides, chemotherapy was also an important treatment for BC, we found that most of BC patients in southern China are treated with chemotherapy, which was approximately 97.01%. But there were only about 24.08% of BC patients had received chemotherapy in SEER cohort (*P* = 0.000), which indicated that there are great differences in BC treatment between China and the United States.

Additionally, with increasing age (from 70’s to 90’s), the proportion of young BC patients in SEER cohort increased gradually, while the proportion of elderly patients decreased gradually. Furthermore, the median age at diagnosis was relativly unchanged, they were 36 year, 56 year and 77 year respectively, which may be related to the early diagnosis of BC.

There are some limitations to this study. The data from southern China cohort are from Yunnan Cancer Hospital, Afiliated Tumor Hospital of Guangxi Medical University and The First People's Hospital of Foshan, these data may therefore be slightly different from that of the National Cancer Registry System. In addition, the presence of missing data and limited follow-up time can be considered weaknesses of this study. Other limitations include the lack of data on surgical procedures, KI-67 statuses and endocrine therapy in SEER cohort, which limited the analysis of their influence on patients’ survival improvements.

## Conclusion

In conclusion, our study suggested that positive lymph node status may cause the difference of morbidity and mortality of BC patients in China. Furthermore, the differences in treatment methods may be the main reason for the differences between China and seer databases.

## Supplementary Information

Supplementary Information.

## Data Availability

The raw data required to reproduce these findings cannot be shared at this time as the data also forms part of an ongoing study.
