# Supplementary material for: A comparison of Chinese multicenter breast cancer database and SEER database
Source: Sci Rep. 2022 Jun 21;12:10395. doi: 10.1038/s41598-022-14573-4 (PMC9213543; doi:10.1038/s41598-022-14573-4)
Supplement: Supplementary file 1 — Supplementary Information. [file 41598_2022_14573_MOESM1_ESM.docx]

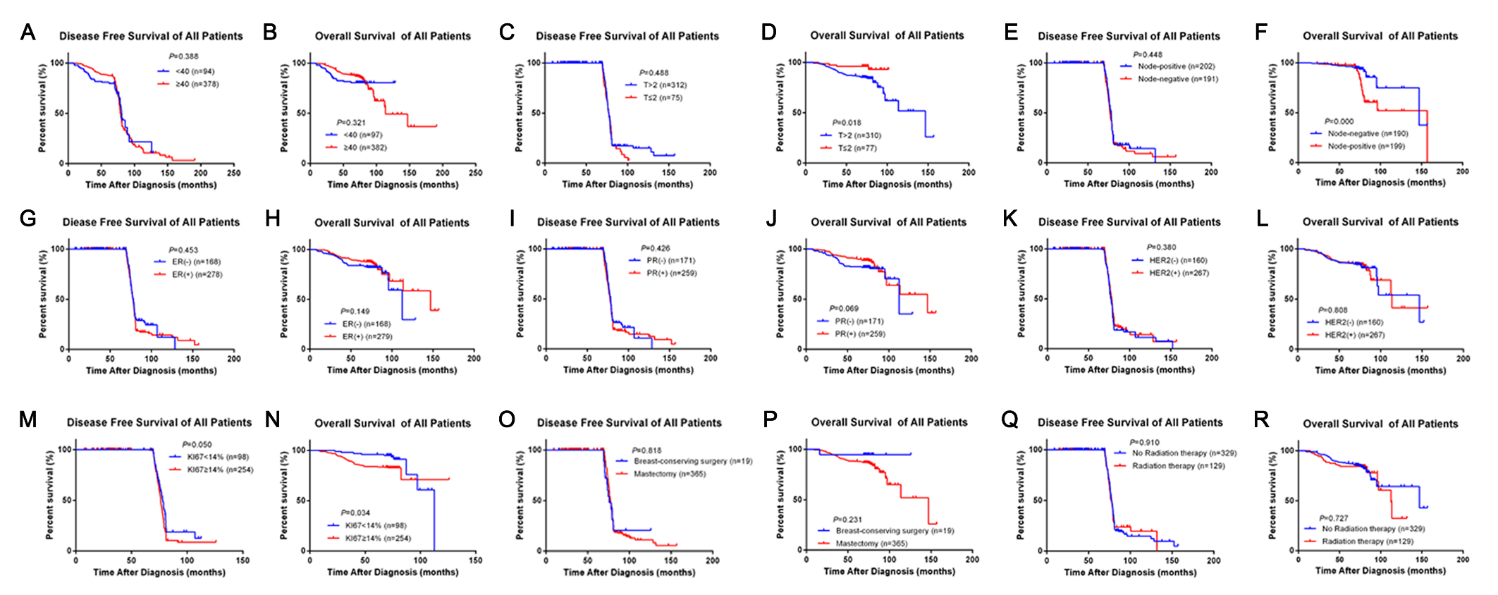


Supplementary figure1: comparison DFS and OS in Southern China under different basic features. (A-B) Kaplan-Meier estimates DFS and OS of all patients in Southern China, (C-D) Kaplan-Meier estimates DFS and OS of different tumour size in Southern China, (E-F) Kaplan-Meier estimates DFS and OS of different nodal stage in Southern China, (G-H) Kaplan-Meier estimates DFS and OS of different ER stage in Southern China; (I-J) Kaplan-Meier estimates DFS and OS of different PR stage in Southern China, (K-L) Kaplan-Meier estimates DFS and OS of different HER2 stage in Southern China, (M-N) Kaplan-Meier estimates DFS and OS of different KI-67 expresssion in Southern China; (O-P) Kaplan-Meier estimates DFS and OS of surgery treatment in Southern China; (Q-R) Kaplan-Meier estimates DFS and OS of radiation in Southern China
